# Horizontal Gene Acquisitions Contributed to Genome Expansion in Insect-Symbiotic *Spiroplasma clarkii*

**DOI:** 10.1093/gbe/evy113

**Published:** 2018-06-01

**Authors:** Yi-Ming Tsai, An Chang, Chih-Horng Kuo

**Affiliations:** Institute of Plant and Microbial Biology, Academia Sinica, Taipei, Taiwan

**Keywords:** mollicutes, *Spiroplasma*, symbiont, comparative genomics, horizontal gene transfer (HGT)

## Abstract

Genome reduction is a recurring theme of symbiont evolution. The genus *Spiroplasma* contains species that are mostly facultative insect symbionts. The typical genome sizes of those species within the Apis clade were estimated to be ∼1.0–1.4 Mb. Intriguingly, *Spiroplasma clarkii* was found to have a genome size that is >30% larger than the median of other species within the same clade. To investigate the molecular evolution events that led to the genome expansion of this bacterium, we determined its complete genome sequence and inferred the evolutionary origin of each protein-coding gene based on the phylogenetic distribution of homologs. Among the 1,346 annotated protein-coding genes, 641 were originated from within the Apis clade while 233 were putatively acquired from outside of the clade (including 91 high-confidence candidates). Additionally, 472 were specific to *S. clarkii* without homologs in the current database (i.e., the origins remained unknown). The acquisition of protein-coding genes, rather than mobile genetic elements, appeared to be a major contributing factor of genome expansion. Notably, >50% of the high-confidence acquired genes are related to carbohydrate transport and metabolism, suggesting that these acquired genes contributed to the expansion of both genome size and metabolic capability. The findings of this work provided an interesting case against the general evolutionary trend observed among symbiotic bacteria and further demonstrated the flexibility of *Spiroplasma* genomes. For future studies, investigation on the functional integration of these acquired genes, as well as the inference of their contribution to fitness could improve our knowledge of symbiont evolution.

## Introduction

The patterns of genome evolution among diverse symbiotic bacteria are characterized by a general trend of genome reduction (Moran and Plague 2004; Ochman and Davalos 2006; Toft and Andersson 2010; McCutcheon and Moran 2012; Moran and Bennett 2014). This observation is likely a combined result of the mutational bias towards deletions commonly observed in bacteria (Mira et al. 2001; Kuo and Ochman 2010), the lack of selection against gene losses in stable and nutrient-rich environments, and the elevated levels of genetic drift due to host restriction (Kuo et al. 2009; Novichkov et al. 2009). Although most free-living bacteria have a genome size that is >4 Mb, symbionts usually have a smaller genome. The most extreme examples of genome reduction were found among those obligate intracellular nutritional mutualists of insects, which have genome sizes in the range of ∼0.1–1.0 Mb (Moran and Bennett 2014).

The genus *Spiroplasma* within the class Mollicutes contains diverse species that are mostly facultative insect symbionts capable of horizontal transmission (Gasparich et al. 2004; Regassa and Gasparich 2006; Gasparich 2010). In recent years, these bacteria have been developed into a model system for the study of symbionts (Anbutsu and Fukatsu 2011; Bolaños et al. 2015; Lo et al. 2016). This paraphyletic genus contains several major clades, with the Apis clade as the most genetically diverse and species-rich one. Based on the genome size estimates by pulsed-field gel electrophoresis (PFGE), most of these *Spiroplasma* species have a genome size of ∼1.0–1.4 Mb (Carle et al. 1995). Given these general observations, it is interesting to note that *Spiroplasma clarkii*, a facultative symbiont residing in the gut of larval/adult Scarabaeidae beetles without apparent effect on its host, was found to have a genome size that is >30% larger than the median of other species within the same clade (Whitcomb et al. 1993). To investigate the evolutionary processes and the genetic changes that led to the genome expansion in this bacterium, we determined the complete genome sequence of *S. clarkii* for comparative analysis.

## Materials and Methods

The procedures for genome sequencing and phylogenetic inference were based on those described in our previous studies (Lo, Chen, et al. 2013; Lo, Ku, et al. 2013; Chang et al. 2014; Lo et al. 2015). The bioinformatics tools were used with the default settings unless stated otherwise. Briefly, the bacterial strain *Spiroplasma clarkii* CN-5^T^ was acquired from the German Collection of Microorganisms and Cell Cultures (catalogue number: DSM 19994^T^). For whole-genome shotgun sequencing, one paired-end library (∼550 bp insert and 430X coverage) and one mate-pair library (∼4.5 kb insert and 60X coverage) were prepared and sequenced using the MiSeq platform (Illumina, USA). The *de novo* assembly was performed using ALLPATHS-LG release 52188 (Gnerre et al. 2011), followed by gap closure and validation using PCR and Sanger sequencing until the complete sequence of the circular chromosome was obtained. The programs RNAmmer (Lagesen et al. 2007), tRNAscan-SE (Lowe and Eddy 1997) and PRODIGAL (Hyatt et al. 2010) were used for gene prediction. The annotation was based on the homologous genes in other *Spiroplasma* genomes (supplementary table S1, Supplementary Material online) as identified by OrthoMCL (Li et al. 2003), followed by manual curation based on the KEGG (Kanehisa et al. 2016) and COG databases (Tatusov et al. 2003).

To identify the homologs of protein-coding genes in other bacteria for each Apis clade species, we performed BLASTP (Camacho et al. 2009) search against the NCBI nonredundant database (version date: March 26, 2018). After removing the self-hit and low-quality hits (i.e., high-scoring pairs accounting for <90% of the query length or amino acid sequence similarity <40%), up to five top hits were collected for each query (supplementary table S2, Supplementary Material online). Representative species from these hits (supplementary table S1, Supplementary Material online) were selected for an additional round of homologous gene identification by OrthoMCL. Putatively acquired islands, defined as regions that have at least five acquired genes and exhibit synteny conservation with species outside of the Apis clade, were identified.

For maximum likelihood phylogenetic analysis, two species phylogenies were inferred. The first one was focused on the Apis clade, the amino acid sequences of the shared single-copy genes were extracted from the OrthoMCL results used for annotation. The multiple sequence alignment was performed using MUSCLE v3.8 (Edgar 2004) for each gene. The concatenated alignment was analyzed using PhyML v3.0 (Guindon and Gascuel 2003). The proportion of invariable sites and the gamma distribution parameter were estimated from the data set, the number of substitute rate categories was set to four. The bootstrap supports were estimated based on 1,000 replicates. The second species phylogeny with broader taxon sampling was based on the BLASTP search result. The 16S rDNA sequences were extracted from the representative species among all hits and processed using the same procedure. For the inference of individual gene trees, we relaxed the criteria for filtering out low quality BLASTP hits (i.e., high-scoring pairs accounting for <80% of the query length or amino acid sequence similarity <30%) in an attempt to identify more distant homologs. Putatively acquired genes in *S. clarkii* with at least five homologs from other Apis clade species among the top 100 hits were selected for phylogenetic analysis using the same procedure. The GenBank accession numbers for all of the sequences included in the phylogenetic analysis are provided in supplementary table S1, Supplementary Material online.

## Results and Discussion

**Fig. 1.—:**
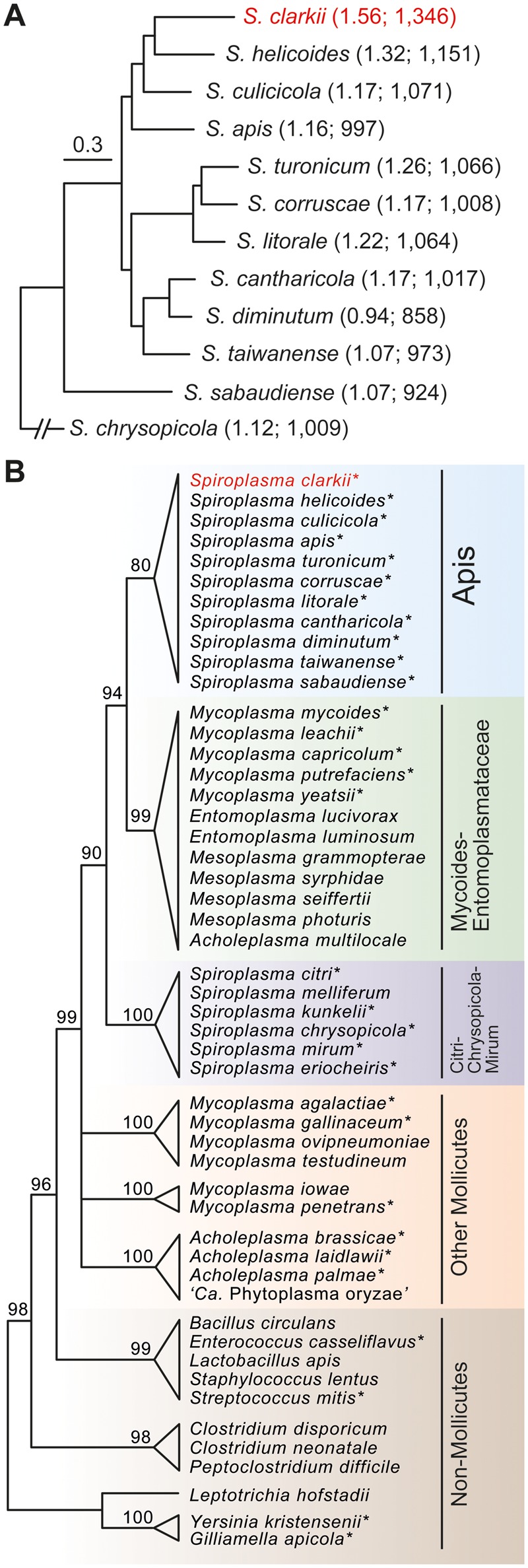
Maximum likelihood phylogenies of representative lineages. (*A*) A phylogram of the Apis clade based on a concatenated alignment of 412 single-copy genes and 148,728 aligned amino acid sites. All nodes received >95% bootstrap support. The numbers in parentheses indicate the chromosome size (unit: Mb) and the number of protein-coding genes. (*B*) A cladogram based on the 16S rDNA phylogeny of the representative species identified in the homologous gene search. The bootstrap support for each major clade is labeled above the branch; internal branches with a bootstrap support below 80% were collapsed. Species highlighted with “*” are those with genome sequences available and included in the identification of horizontally transferred gene islands. All accession numbers are provided in supplementary table S1, Supplementary Material online.

The complete genome sequence of *S. clarkii* contains one circular chromosome that is 1.56 Mb in size; no plasmid was found. Although this size is 12% smaller than the 1.77 Mb estimate based on PFGE (Whitcomb et al. 1993), it is still >30% larger than the median of other Apis clade species with complete genome sequences available (fig. 1*A*). Moreover, comparison between the actual genome size of these species with previous estimates (Carle et al. 1995; Williamson et al. 1996; Whitcomb et al. 1997; Hélias et al. 1998) revealed that the PFGE method typically overestimates the genome size by ∼10–15%.

**Fig. 2.—:**
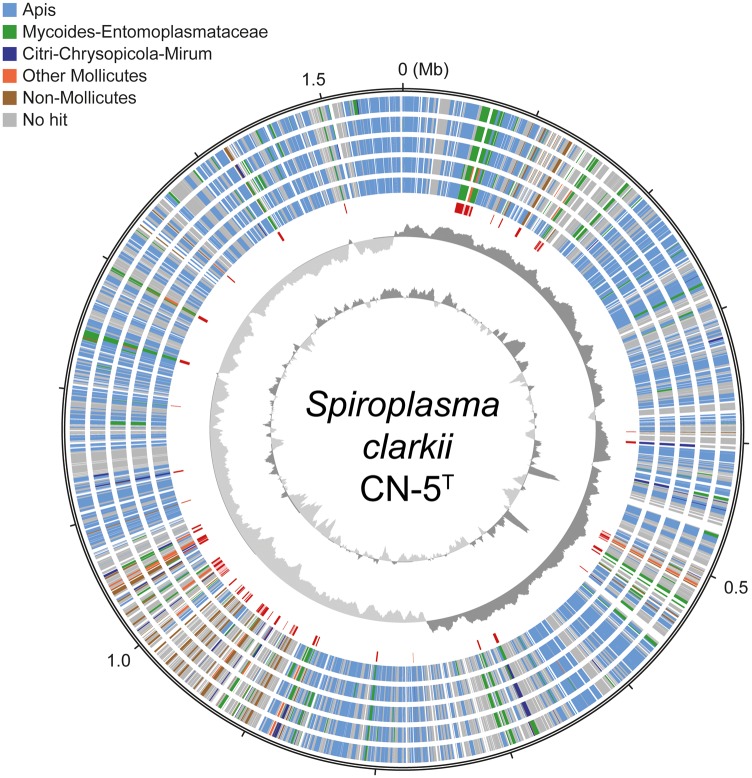
Chromosomal organization of *S. clarkii*. Concentric circles from the inside out: 1) GC content (above average: dark gray; below average: light gray; two high GC peaks near the 0.5 Mb mark correspond to the rRNA gene clusters), 2) GC skew (positive: dark gray; negative: light gray), 3) locations of the high-confidence candidates of acquired genes (colored in red; putatively acquired islands with at least five genes from the same donor are highlighted with a pink background), 4–8) phylogenetic affiliations of the top five BLASTP hits from the NCBI nonredundant database (color-coded according to the legend), and 9) scale marks (unit: Mb).

Examination of the chromosome organization and gene content (fig. 2) revealed that the genome expansion was not attributed to the invasion of plectroviruses as those found within the *Spiroplasma* Citri clade, in which viral sequences account for ∼20% of the chromosome in extant species (Carle et al. 2010; Alexeev et al. 2012; Ku et al. 2013; Lo, Chen, et al. 2013; Paredes et al. 2015). Rather, acquisition of protein-coding genes through horizontal gene transfer (HGT) appeared to be a major factor. Among the 1,346 annotated protein-coding genes, 641 (48% of the gene count and 45% of the chromosome length) were inferred as being originated from within the Apis clade (table 1). For these genes, the top five BLASTP hits within the NCBI nonredundant database did not involve any organism outside of the Apis clade, which suggested that these genes were either inherited vertically or at least did not involve recent HGT from donors outside of the Apis clade. There are 472 species-specific genes (i.e., those without any identifiable homolog in the current database), which correspond to 35% of the gene count and 29% of the chromosome length. These species-specific genes appear to be the main contributing factor of the genome expansion observed in *S. clarkii* (table 1). Although some of these may be artifacts of gene prediction, we expect that a high proportion of these genes may be acquired genes without known donors. The reason for this inference is that all of these *Spiroplasma* genomes were annotated by our research group based on the same procedure and on average those species-specific genes account for only ∼16% of the total gene count in other Apis clade species. Unfortunately, although the hypothesis that most of these species-specific genes were acquired through HGT is plausible, direct evidence for or against this hypothesis is lacking. The remaining genes were assigned to two classes of HGT candidates, including 142 low-confidence ones (i.e., the top five hits involved a mixture of species from within the Apis clade and other more divergent ones; 11% of the count and 9% of the length) and 91 high-confidence ones (i.e., the top five hits did not involve any Apis clade species; 7% of the count and 6% of the length). The low confidence candidates may include those acquired prior to the divergence between *S. clarkii* and *Spiroplasma helicoides*, or those with more complex history such as multiple gain/loss events. However, due to the limited number of homologs available in the current database, as well as the finding that many of these low confidence candidates correspond to short hypothetical proteins, it is difficult to infer the exact evolutionary history of these genes. Furthermore, because those low confidence candidates account for ∼10% of total gene count in other Apis clade species as well, these genes are unlikely to be a main contributing factor of genome expansion in *S. clarkii*.

**Table 1:** Classification of Protein-Coding Genes by Putative Origins

Genome	Apis	Species-Specific	HGT-Low Confidence	HGT-High Confidence
*S. clarkii*	641 (48%)	472 (35%)	142 (11%)	91 (7%)
*S. helicoides*	679 (68%)	198 (20%)	99 (10%)	21 (2%)
*S. culicicola*	709 (66%)	228 (21%)	98 (9%)	36 (3%)
*S. apis*	728 (63%)	281 (24%)	109 (10%)	33 (3%)
*S. turonicum*	780 (73%)	149 (14%)	115 (11%)	20 (2%)
*S. corruscae*	667 (69%)	218 (22%)	70 (7%)	18 (2%)
*S. litorale*	789 (74%)	151 (14%)	105 (10%)	21 (2%)
*S. cantharicola*	745 (73%)	123 (12%)	104 (10%)	45 (4%)
*S. diminutum*	770 (76%)	119 (12%)	115 (11%)	4 (0%)
*S. taiwanense*	728 (85%)	50 (6%)	67 (8%)	13 (2%)
*S. sabaudiense*^a^	243 (26%)	338 (37%)	178 (19%)	165 (18%)

Note.—Values indicate the gene count; the percentage of total is provided in parentheses.

a Due to the high level of sequence divergence from other Apis clade species, as well as its basal placement in the species phylogeny, this approach of utilizing BLASTP searches to classify the putative origin of genes is not applicable to *S. sabaudiense*. These values are provided for reference only and are not included in the calculation of average percentages among Apis clade species as discussed in the main text.

**Fig. 3.—:**
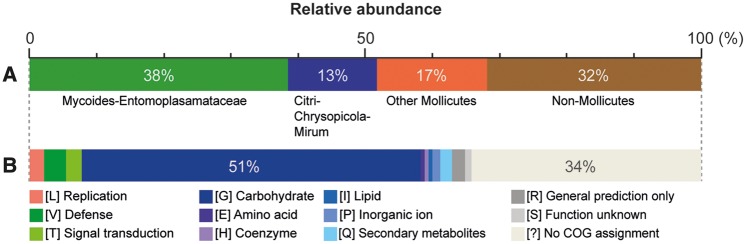
Classification of the high-confidence candidates of acquired genes. (*A*) Phylogenetic assignments of the most likely donors. (*B*) Functional assignments according to the COG categories.

Compared with other Apis clade species, the number and proportion of high-confidence HGT candidates are both much higher in *S. clarkii* (table 1). This finding further suggested that gene acquisition is a major contributing factor of the genome expansion. Among those 91 high-confidence candidates, 38% were inferred as originated from the sister Mycoides-Entomoplasmataceae clade based on the phylogenetic distribution of homologs, whereas those from the Citri-Chrysopicola-Mirum clade and other more divergent lineages within the class Mollicutes account for 13% and 17%, respectively (figs. 1*B* and 3*A*). Using individual gene phylogenies for the test of HGT hypothesis was not feasible for most of these candidates; 61 out of these 91 candidates did not have any identifiable homolog in other Apis clade species. Although multiple independent losses in all other Apis clade lineages may also explain the pattern and argue for vertical inheritance, such alternative hypothesis is less parsimonious compared with HGT. For the nine candidates with at least five homologs in other Apis clade species, we inferred the individual gene trees to investigate their evolutionary history (supplementary fig. S1, Supplementary Material online). At least four of these gene trees provided strong support for the HGT hypothesis.

Because the vast majority of Entomoplasmataceae/Spiroplasmataceae species are affiliated with insect hosts for at least a part of their life cycle (Gasparich et al. 2004; Regassa and Gasparich 2006; Gasparich 2010; Gasparich 2014), this overlap in ecological niche could have promoted the HGT events among these lineages. Moreover, despite the phylogenetic divergence, all these Mollicutes lineages share the same alternative genetic code (i.e., UGA changed from stop to tryptophan) and a strong nucleotide composition bias toward A + T. These shared genomic traits could have promoted the retention and integration of those acquired genes (Lo and Kuo 2017). The chromosomal region at ∼0.9–1.1 Mb appeared to be the major hot spot for foreign genes (fig. 2). A total of 45 genes were identified in seven islands with at least five acquired genes from the same donor with synteny conservation. Interestingly, most of the gene acquisitions did not disrupt the patterns of GC skew and gene orientation (fig. 2), suggesting that those that did may be selected against.

**Fig. 4.—:**
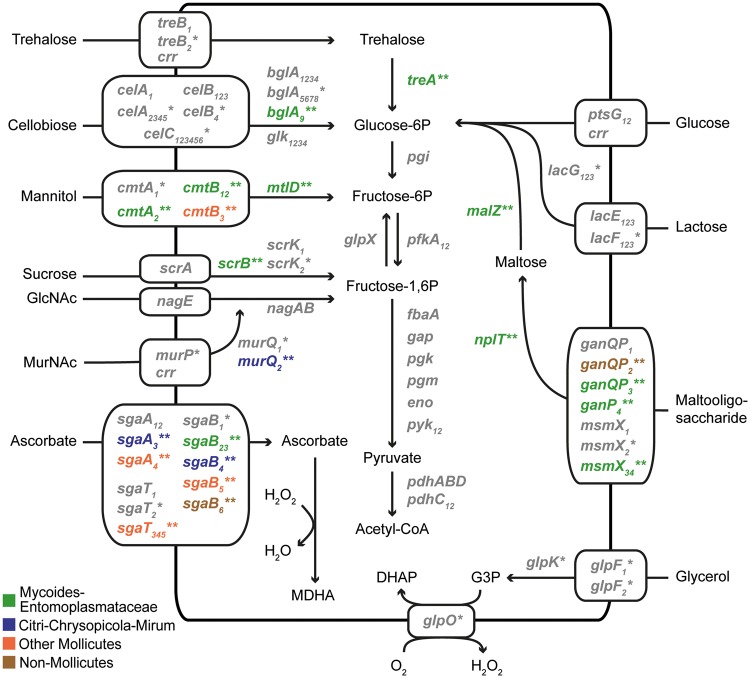
Carbohydrate transport and metabolism. Genes that are putatively involved in horizontal transfer are highlighted by “*” (low-confidence) or “**” (high-confidence; color-coded according to the putative donors). For multicopy genes, subscripts following the gene name are used to distinguish different copies. Abbreviations: DHAP, dihydroxyacetone phosphate; G3P, glycerol-3-phosphate; GlcNAc, N-Acetylglucosamine; MDHA, monodehydroascorbate; MurNAc, N-acetylmuramic acid.

Regarding the functions, carbohydrate transport and metabolism is the most dominant category that accounts for 51% of those high-confidence candidates (fig. 3*B*). This finding is worth noting because carbohydrate metabolism is highly variable among *Spiroplasma* species (Chang et al. 2014; Lo et al. 2015) and is important in their physiology and ecology (Regassa and Gasparich 2006; Gasparich 2010). Moreover, carbohydrate metabolism genes are often involved in HGT and have been shown to be integrated into the gene expression regulation in other Apis clade species (Lo and Kuo 2017). Intriguingly, extensive gene acquisition has also been reported for *Spiroplasma eriocheiris* of the Mirum clade, in which ∼7% of the genes may be acquired from non-*Spiroplasma* donors (Lo et al. 2015). However, while most of the acquired genes in *S. eriocheiris* correspond to novel transporters and pathways, HGT in *S. clarkii* mostly contributed to the copy number expansion of existing genes (fig. 4). The explanation for this difference between those two species is unclear.

## Conclusion

The findings of this work provided an interesting case against the general evolutionary trend of genome reduction observed among symbiotic bacteria and further demonstrated the flexibility of *Spiroplasma* genomes. For future studies, investigations on the fitness effects of these gene acquisitions, as well as expanding taxon sampling to investigate the generality of genome expansion in different bacteria could further improve our knowledge of symbiont evolution.

## Supplementary Material

Supplementary data are available at *Genome Biology and Evolution* online.

## Supplementary Material

Supplementary Data
