# Supplementary material for: Horizontal Gene Acquisitions Contributed to Genome Expansion in Insect-Symbiotic Spiroplasma clarkii
Source: Genome Biol Evol. 2018 Jun 1;10(6):1526–32. doi: 10.1093/gbe/evy113 (PMC6007557; doi:10.1093/gbe/evy113)

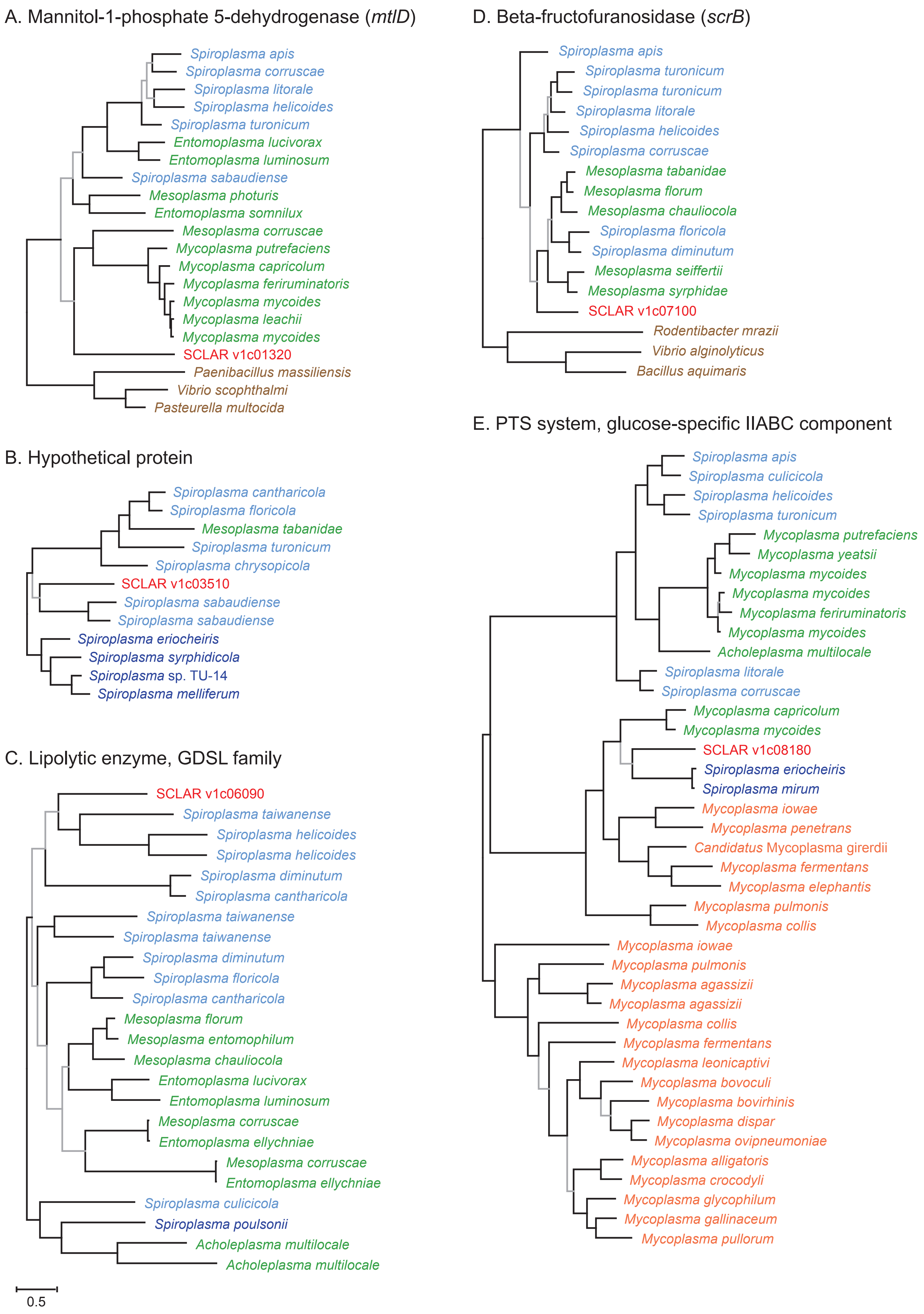
A. Mannitol-1-phosphate 5-dehydrogenase (*mtlD*)
Spiroplasma apis
Spiroplasma corruscae
Spiroplasma litorale
Spiroplasma helicoides
Spiroplasma turonicum
Entomoplasma lucivorax
Entomoplasma luminosum
Spiroplasma sabaudiense
Mesoplasma photuris
Entomoplasma somnilux
Mesoplasma corruscae
Mycoplasma putrefaciens
Mycoplasma capricolum
Mycoplasma feriruminatoris
Mycoplasma mycoides
Mycoplasma leachii
Mycoplasma mycoides
SCLAR v1c01320
Paenibacillus massiliensis
Vibrio scophthalmi
Pasteurella multocida
B. Hypothetical protein
Spiroplasma cantharicola
Spiroplasma floricola
Mesoplasma tabanidae
Spiroplasma turonicum
Spiroplasma chrysopicola
SCLAR v1c03510
Spiroplasma sabaudiense
Spiroplasma sabaudiense
Spiroplasma eriocheiris
Spiroplasma syrphidicola
Spiroplasma sp. TU-14
Spiroplasma melliferum
C. Lipolytic enzyme, GDSL family
SCLAR v1c06090
Spiroplasma taiwanense
Spiroplasma helicoides
Spiroplasma helicoides
Spiroplasma diminutum
Spiroplasma cantharicola
Spiroplasma taiwanense
Spiroplasma taiwanense
Spiroplasma diminutum
Spiroplasma floricola
Spiroplasma cantharicola
Mesoplasma florum
Mesoplasma entomophilum
Mesoplasma chauliocola
Entomoplasma lucivorax
Entomoplasma luminosum
Mesoplasma corruscae
Entomoplasma ellychniae
Mesoplasma corruscae
Entomoplasma ellychniae
Spiroplasma culicicola
Spiroplasma poulsonii
Acholeplasma multilocale
Acholeplasma multilocale
0.5
D. Beta-fructofuranosidase (*scrB*)
Spiroplasma apis
Spiroplasma turonicum
Spiroplasma turonicum
Spiroplasma litorale
Spiroplasma helicoides
Spiroplasma corruscae
Mesoplasma tabanidae
Mesoplasma florum
Mesoplasma chauliocola
Spiroplasma floricola
Spiroplasma diminutum
Mesoplasma seiffertii
Mesoplasma syrphidae
SCLAR v1c07100
Rodentibacter mrazii
Vibrio alginolyticus
Bacillus aquimaris
E. PTS system, glucose-specific IIABC component
Spiroplasma apis
Spiroplasma culicicola
Spiroplasma helicoides
Spiroplasma turonicum
Mycoplasma putrefaciens
Mycoplasma yeatsii
Mycoplasma mycoides
Mycoplasma mycoides
Mycoplasma feriruminatoris
Mycoplasma mycoides
Acholeplasma multilocale
Spiroplasma litorale
Spiroplasma corruscae
Mycoplasma capricolum
Mycoplasma mycoides
SCLAR v1c08180
Spiroplasma eriocheiris
Spiroplasma mirum
Mycoplasma iowae
Mycoplasma penetrans
Candidatus Mycoplasma girerdii
Mycoplasma fermentans
Mycoplasma elephantis
Mycoplasma pulmonis
Mycoplasma collis
Mycoplasma iowae
Mycoplasma pulmonis
Mycoplasma agassizii
Mycoplasma agassizii
Mycoplasma collis
Mycoplasma fermentans
Mycoplasma leonicaptivi
Mycoplasma bovoculi
Mycoplasma bovirhinis
Mycoplasma dispar
Mycoplasma ovipneumoniae
Mycoplasma alligatoris
Mycoplasma crocodyli
Mycoplasma glycophilum
Mycoplasma gallinaceum
Mycoplasma pullorum

F. PTS system, glucose-specific IIABC component

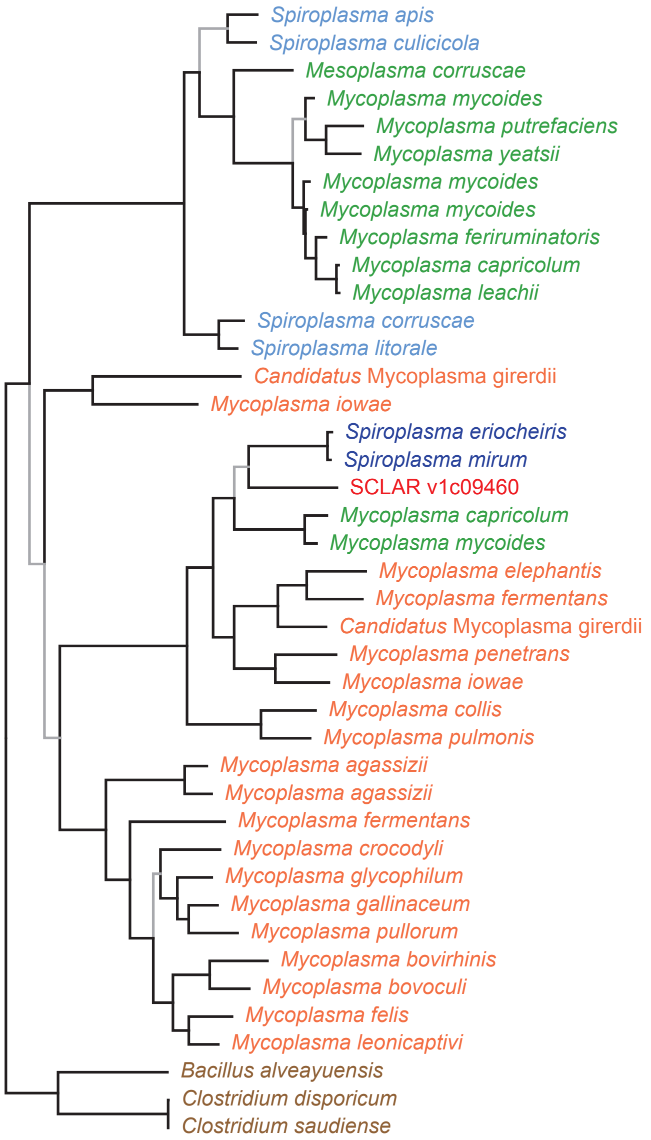

G. 6-phospho-beta-glucosidase (*bglA*)

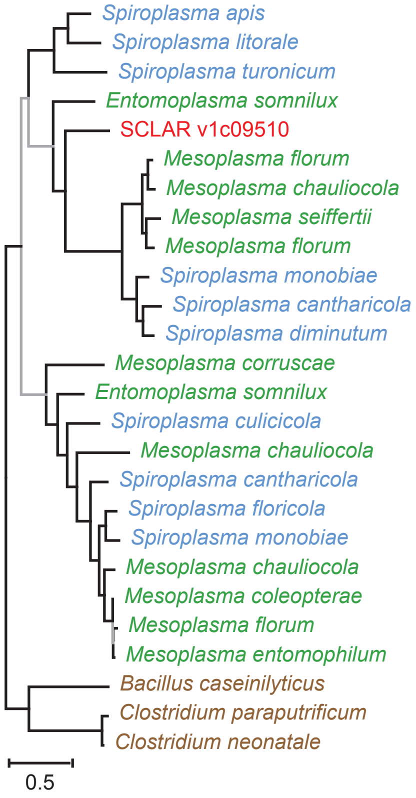

H. Cation-transporting ATPase (*ctp*)

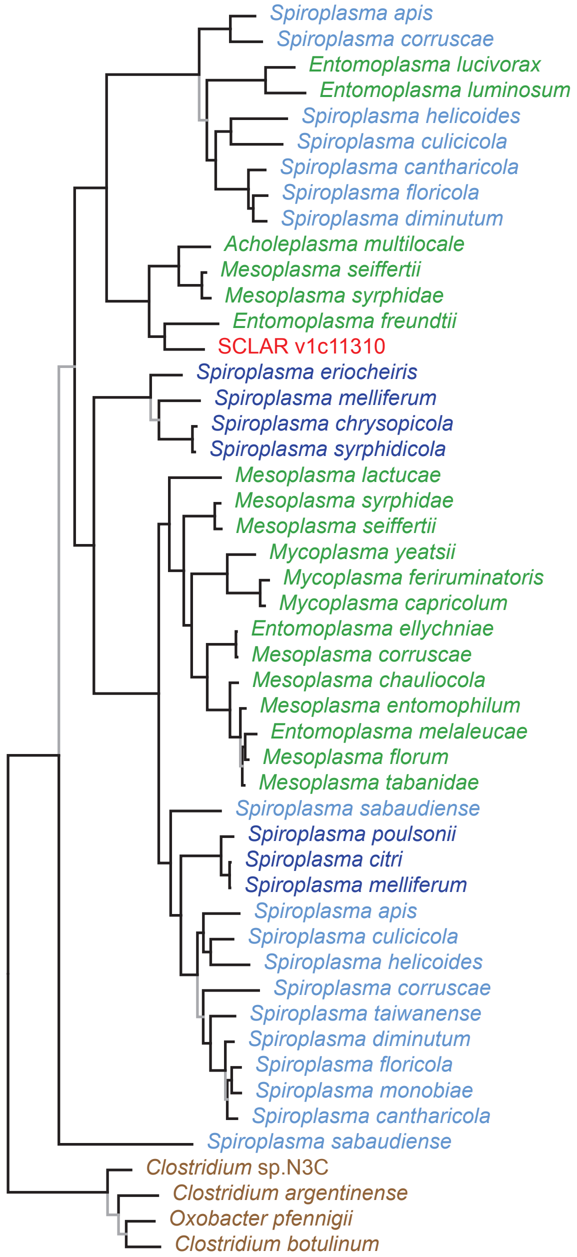

I. aldo/keto reductase

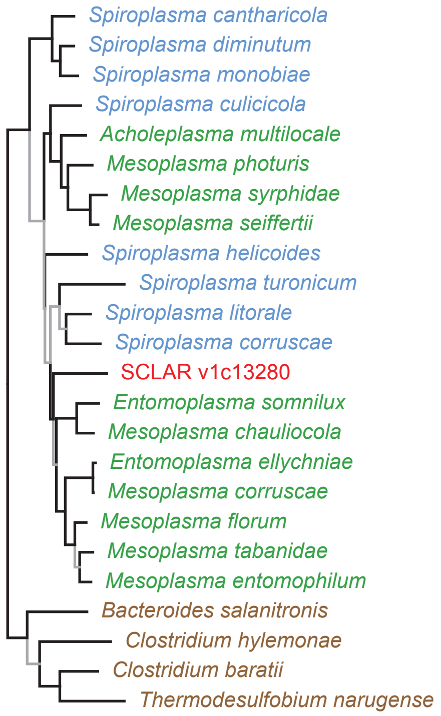

Supplement: Supplementary Data [file evy113_suppl.zip › fig.genetree.v7.pdf]
